# Retraction: Molecular Pathology in Modern Medicine: A Review of Genomic, Proteomic, and Epigenetic Insights Into Disease Mechanisms

**DOI:** 10.7759/cureus.r222

**Published:** 2026-03-27

**Authors:** Santosh Jayant, Nithi Doley, N. Swapna, Hari Prasad, Kumar Sambhav, Baijnath Das

**Affiliations:** 1 Department of Pathology and Laboratory, Amaltas Institute of Medical Science, Amaltas University, Dewas, IND; 2 Department of Pathology, Neelima Institute of Medical Sciences, Hyderabad, IND; 3 Department of Pathology, Aarupadai Veedu Medical College and Hospital, Vinayaka Missions Research Foundation (Deemed to be University), Puducherry, IND; 4 Department of Microbiology, Sree Balaji Medical College, Bharath Institute of Higher Education and Research, Chennai, IND; 5 Department of Anatomy, All India Institute of Medical Sciences, Bilaspur, IND; 6 Department of Medical Laboratory Technique, Teerthanker Mahaveer University, College of Paramedical Sciences, Moradabad, IND

This article has been retracted by the Editor-in-Chief due to suspicion of academic fraud. The same manuscript was submitted separately by a different group of authors prior to this article's publication. Neither group of authors could provide an adequate explanation as to how they both came to have the exact same manuscript.

